# Aging and longevity in decades‐old genebanked seeds from U.S. endangered plant species: Assessments using survival and RNA integrity assays

**DOI:** 10.1002/ajb2.70169

**Published:** 2026-02-24

**Authors:** Christina Walters, Katherine D. Heineman, Lisa Hill, Hannah Tetreault, Parker Tyler, Zoe Zingerman, Shaimaa Ibrahim, Joyce Maschinski

**Affiliations:** ^1^ USDA‐ARS National Laboratory for Genetic Resources Preservation 1111 South Mason Street Fort Collins 80521 Colorado USA; ^2^ Center for Plant Conservation 15600 San Pasqual Valley Road Escondido 92027 California USA; ^3^ San Diego Zoo Wildlife Alliance, Conservation Science 15600 San Pasqual Valley Road Escondido 92027 California USA

**Keywords:** genebank, germination, long‐term storage, RIN, RNA integrity, seed aging, seed dormancy, seed longevity, seed storage, viability assay, wild species

## Abstract

**Premise:**

Seed longevity is critical for successful genebanking, but it is hard to detect or predict. We examined survival of genebanked seeds from species native to the United States to estimate longevity. We tested whether RNA integrity (RIN) can be used to detect aging and predict mortality.

**Methods:**

Dry seeds from >100 species were stored for 28 ± 7 yr at −18°C. A recently harvested sample (cohort) from the same population provides a zero‐time reference. Germination and RIN were assessed and differences between cohorts were used to distinguish short‐lived seeds from long‐lived seeds.

**Results:**

No differences in germination or RIN were detected between cohorts in about one‐fourth of the species. Viability and/or RIN was lower in the stored cohort than in the recently harvested cohort in most species, and the size of the difference was used to infer aging rates. Differences in germination and RIN were correlated among the 100 samples tested; moderate correlation coefficients indicate that additional factors are involved in seed aging and its detection.

**Conclusions:**

Overall, longevity in the genebank appears to be similar for seeds from wild and domesticated species. We identified species that appeared to produce quite long‐lived and short‐lived seeds. Seeds from wild species tend to germinate slowly and asynchronously, and this confounds comparisons across storage times; deterioration is detected mostly after severe mortality. By contrast, RIN values decline before viability loss is detected and appear to be unaffected by wild seed traits. RIN tests during early storage can help predict seed longevity.

Seed longevity—the duration that seeds survive in storage or in soil—affects commercial seed production, genebanking, weed emergence, and post‐disturbance land recovery. Exploration of interspecific differences in seed longevity has prompted numerous surveys of seed performance under various storage conditions (Priestley et al., 1985; Roos and Davidson, 1992; Walters et al., 2005a; Pérez‐García et al., 2007; Nagel et al., 2009; Probert et al., 2009; Mondoni et al., 2011; Merritt et al., 2014; Desheva, 2016; Davies et al., 2020; Yamasaki et al., 2020; Gianella et al., 2022; Niñoles et al., 2022), some “legacy” experiments (e.g., Went and Munz, 1949; Walters et al., 2004; Fleming et al., 2019, 2023), and several meta‐analyses of surveys (Hay and Probert, 2013; Colville and Probert, 2019; Solberg et al., 2020). Evidence of very long‐lived seeds is exciting (Priestley and Posthumus, 1982; Shen‐Miller et al., 1995; Steiner and Ruckenbauer, 1995; Daws et al., 2007; Yashina et al., 2012; Gros‐Balthazard et al., 2021), and the reemergence of species thought to have been extirpated from a locale attests to the resiliency of natural systems despite human intervention (Kiss et al., 2018; Shiferaw et al., 2018). Here, we report seed survival from some of the earliest efforts to genebank seeds from wild species. The U.S.‐based Center for Plant Conservation (CPC) began genebanking in 1984, targeting plant species in need of ex situ conservation (Kennedy, 2008). These efforts offer a unique data set to document the effects of long‐term storage on a diverse array of seeds from wild species.

The innate ability of dry seeds to survive for decades inspired plant genebanking initiatives in the early 2000s, thanks to the Millennium Seed Bank Partnership (Hay and Probert, 2013; Wambugu et al., 2023) and the Crop Diversity Trust (Hawtin and Fowler, 2012). On the basis of research using crops, seeds were termed “orthodox” if survival time increased as storage conditions became progressively drier and colder. In the 1970s and 1980s, the advantage of longer shelf life prompted several agriculturally based genebanks to reduce storage temperatures from refrigerated (or even uncontrolled temperature) to freezer conditions; this major switch in storage facilities and protocols extended seed life spans by decades (Walters et al., 2005a; Nagel et al., 2009; Desheva, 2016; Yamasaki et al., 2020; Guzzon et al., 2021; Gianella et al., 2022) and became a key feature of international standards for long‐term storage of orthodox seeds (FAO, 2014). In the early 1990s, there was some pessimism that these emerging technologies would be embraced by the conservation community and applied effectively for ex situ conservation of wild plant species (excluding seeds from wild congeners of crops; e.g., Soulé, 1991). Effective genebanking is highly technical, and filling the knowledge gaps about seed germination and storage behavior is difficult for wild species because the seeds are intrinsically scarce, heterogeneous, and unpredictable (De Vitis et al., 2020; Wambugu et al., 2023). There are few reports describing performance of wild seeds in genebanks, with some notable exceptions (Pérez‐García et al., 2007; Ballesteros and Pence, 2019; Sommerville et al., 2021; Gianella et al., 2022).

Most of our knowledge of wild seed longevity comes from aging simulations conducted at high humidity and temperature (e.g., Probert et al., 2009; Mondoni et al., 2011; Merritt et al., 2014; Davies et al., 2020; Niñoles et al., 2022; Sommerville et al., 2023). Degradation occurs within days to weeks during simulated aging, making this a compelling experimental approach, compared with the decades needed to detect change under genebanking conditions (e.g., Walters et al., 2005; Pérez‐García et al., 2007; Nagel et al., 2009; Desheva, 2016; Yamasaki et al., 2020; Gianella et al., 2022). Simulated aging is used primarily to make comparisons of longevity among genetic lines (e.g., Rajjou et al., 2008; Hang et al., 2015; Saighani et al., 2021; Gianella et al., 2022; Zhang et al., 2022; Pirredda et al., 2023), by assuming that moisture effects are constant within a species (Ellis and Roberts, 1980), which can be an arguable point (e.g., Niedzielski et al., 2009; Gianella et al., 2022). We know that moisture effects vary among species; thus, inferences about relative longevity among diverse species in a genebank based on simulated aging studies should, at least, have support from dry storage or actual genebanking data. Another goal of simulated aging studies is to provide a timeframe for survival under genebanking conditions. Few studies link timeframes between simulated aging and dry storage approaches, simply because the principles for these extrapolations are poorly understood. Briefly, these principles reside in structural changes (solid ↔ fluid) in seed cytoplasm that occur between 50% and 65% relative humidity (at room temperature), depending on chemical composition and molecular organization (Sun and Leopold, 1997; Walters, 2015; Ballesteros et al., 2017; Ballesteros et al., 2020; Walters et al., 2020; Zinsmeister et al., 2020).

The concept of short‐lived and long‐lived seeds among different species is not novel (Ewart, 1908), and various famous studies have documented seed longevity differences among species (Went and Munz, 1949; Priestley et al., 1985; Fleming et al., 2023; SER, 2023). Some studies show variation among seed lots within a species (e.g., Walters et al., 2005a; Nagel et al., 2009; Niedzielski et al., 2009; Lee et al., 2019; Guzzon et al., 2021; Saighani et al., 2021; Gianella et al., 2022; Niñoles et al., 2022; Selvarani et al., 2025; Tetreault et al., 2024). The source of the variation is mostly unexplained; there appears to be no correlation with seed size, seed outer coverings, or accumulated food reserves. In the cross‐cutting field of dry biological systems, molecules that stabilize solidifying structures appear to be key but sequestration of vulnerable molecules and inhibition of chemical reactivity may also be involved (Marks et al., 2025).

The eventual cause of death is basic to studies of longevity. Dry seeds do not show symptoms of aging per se (i.e., subtle changes that indicate progressive loss of functionality), and “aging” is expressed almost exclusively in terms of mortality. Mortality is measured by hydrating seeds and counting the proportion of seeds that have lost metabolic or germination capacity; depending on that proportion, we often refer to a sample as “aged” or “not aged yet.” A time course of “aging” is thus depicted by a sigmoidal curve, indicating an initial lag period which reflects few detected changes followed by a sharper slope that reflects simultaneous deaths of seeds in the sample. Death is caused by changes that occur in the lag phase, but applying cause/effect relationships is difficult when the effect (i.e., death) occurs well after the fact. Oxidative reactions, leading to molecular fragmentation or cross‐linking, are implicated (Bailly et al., 2008; Sano et al., 2016; Nagel et al., 2019; Wiebach et al., 2020; Zinsmeister et al., 2020; Niñoles et al., 2022; Nadarajan et al., 2023; Pirredda et al., 2023). It follows that the duration of survival (i.e., longevity) correlates with the rate at which chemical reactions occur, though such mechanistic studies are extremely rare for dry seeds, which take many years to die.

Emerging analytical techniques allow greater characterization of chemical change within dry biological systems. Increase of intracellular redox, emission of volatile molecules, fragmentation of RNA, and changes to physical properties of lipids can be detected before seeds lose viability. In the present study, we further explore the association between RNA integrity (RIN) and seed longevity. RNA increasingly fragments during seed storage and appears to reflect within‐species variation in seed longevity (Kranner et al., 2011; Fleming et al., 2017, 2019; Zhao et al., 2020; Saighani et al., 2021; Tetreault et al., 2023, 2024). RNA can be extracted from dry seeds, which eliminates some confounding factors when testing metabolism or germination capacity.

This study builds upon previous reports about cohort pairs from wild species that showed overall lower viability (germination proportion, germination speed, and vital staining) and RIN in seeds stored for an average of 28 yr than in recently harvested counterparts (Tetreault et al., 2025; Walters et al., 2025). We hypothesized that seed longevity in the ~100 wild species in our study would be comparable to that reported for genebanked seeds from domesticated species. Comparisons between cohorts provided a means to assess evidence of aging and to identify samples that appeared to be shorter‐ or longer‐lived. We hypothesized that these apparent differences in longevity would correlate with differences in RIN between cohorts and offer the potential to use RIN to detect aging before mortality and to predict longevity.

## MATERIALS AND METHODS

### Source material

We obtained seeds from the CPC's National Collection of rare or endangered plants (Appendix S1: Table S1) that have been placed in safe keeping in genebanks at U.S. botanical gardens or at USDA's National Laboratory for Genetic Resources Preservation (NLGRP). We selected >100 species, representing 39 botanical families, for study to give a broad perspective of seed behavior from diverse botanical backgrounds and habitats. To study seeds that were stored for ≥15 yr, we selected samples that were placed into storage between 1983 and 2010 (referred to as stored seeds or cohorts). To allow for paired comparisons between recently harvested and stored cohorts, a second group of seeds were harvested in 2021–2024 from the same wild population or were regenerated in greenhouses from progeny of that population (referred to as recently harvested cohorts; Appendix S1: Table S2). Average harvest years for stored and recently harvested samples were 1995 and 2022, respectively.

Seeds from the stored cohort were placed under conditions that mostly reflect international recommendations for genebanking—environments at low relative humidity (RH) and −18°C (FAO, 2014)—though eight samples were placed under refrigerated (5°C) and/or cryogenic (around −180°C) conditions. For some of these samples, another sample collected from a nearby population was stored in the freezer for a similar duration, and this made it possible to account for storage temperature. For a different subset of 12 species, initial and monitor data from conspecific seeds harvested between 2005 and 2017 from nearby populations provide intermediate time points to more closely examine the effect of storage time.

Subsamples of seeds were tested for viability using germination assays as described previously (Walters et al., 2025). Most seeds were small and planted on blue blotter paper (Anchor Paper, St. Paul, Minnesota, USA) in Petri plates dampened with deionized water. Larger seeds were rolled in paper towels (Anchor Paper). Seeds that resisted water uptake (i.e., did not swell) were clipped. Some seeds with low germination proportions were planted in damp sphagnum, peat, or soil (Kapecute, Guangzhou, China; purchased through Amazon or from Sun Gro Horticulture, Agawan, Massachusetts, USA). If germination requirements were unknown, a series of treatments, reminiscent of the “move‐along” approach (Baskin and Baskin, 2003), was used in small batches, and comparable results reflecting the most successful treatments were pooled (Walters et al., 2025). Most seeds were placed at temperatures that alternated between 15°C and 25°C or between 20°C and 30°C in 12 hr cycles, with or without prior stratification at 5°C for a few weeks or months (Walters et al., 2025).

Seeds were evaluated for germination (radicle protrusion from covering layers) at weekly or monthly intervals, depending on whether they had recently been moved to a different temperature regimen or rehydrated with a 200 ppm solution of gibberellic acid (GA_3_). Germination tests were considered complete when all the seeds in the sample either germinated, molded, became squishy, appeared to be unfilled (no embryo), or were tested for viability using the vital stain 2,3,5‐triphenyl tetrazolium chloride (TZ) (Miller, 2005). The median duration for an assay was 144 d, with minimum and maximum durations of 3 d and 807 d. Varying treatments sometimes yielded high germination proportions, and when germination proportion among assays was within 0.2 of the highest value, we pooled the number of germinated and sown seeds for each species × cohort treatment in order to maximize the number of seeds accounted for in an assay (i.e., a median of 75 seeds and minimum of 24).

Given sufficient seed availability, TZ tests were conducted on both cohorts if one cohort exhibited a germination proportion <0.5 and seeds were large enough to dissect (i.e., >0.8 mg/seed; Walters et al., 2025). Subsamples of 20–30 seeds were placed on blue blotter paper in Petri plates and dampened with deionized water overnight. Seeds were then cut to reveal the presence/absence of an embryo (filled vs. unfilled), and viability of embryos was further assessed by immersing cut seeds in 1% TZ solution (Alfa Aesar, Thermo Fisher Scientific, Waltham, Massachusetts, USA) at 25°–35°C for 1 d. Seeds were assessed as viable or inviable on the basis of perceived respiratory activity (red staining) in vital embryonic tissues (Miller, 2005).

The proportion of unfilled seeds (i.e., seeds lacking embryos) in a sample affects comparisons between cohorts and among species. Therefore, we needed to account for unfilled seeds in germination and viability assays. Proportion of unfilled seeds in the seed lot was estimated by combining results of all germination and TZ assays and calculated as the sum of identified unfilled seeds divided by the number of seeds sown or hydrated for different assays (Crawley, 2012). Germination proportion (germ) was then expressed as the total number of germinating seeds for optimized germination conditions divided by the total number of *filled* seeds (Crawley, 2012), with the denominator calculated from total number of seeds sown (range: 24–150; average ± SD: 70 ± 48) minus the number of unfilled seeds in the assays. This treatment tends to overestimate the viability of a sample when the proportion of unfilled seeds is ≥ 0.3, but it provides a more realistic comparison of viability between cohorts.

### RNA integrity

RNA was extracted from seeds of recently harvested and stored cohorts, using methods described in Tetreault et al. (2025). When seeds were sufficiently large (>0.8 mg each), unfilled seeds were removed and RNA was extracted only from filled seeds. Moreover, thick seed coverings were peeled off and discarded from large seeds, such that RNA was extracted mostly from embryos. In the few cases where >98% of the seeds were empty, a sample comprised of degraded internal tissues was prepared with lab notes alerting to probable extraction failure. Between 1 mg and 66 mg (average = 12.3 ± 6.2 mg) of seed tissue was used for each biological replicate. Seed tissues were ground to a fine powder in the presence of ~1 mg polyvinylpyrrolidone‐40 (PVP; Fisher Scientific, Fair Lawn, New Jersey, USA). RNA was extracted using the Plant RNeasy kit (Qiagen, Hilden, Germany) and Nucleospin RNA kit with Fruit‐mate (Takara, Düren, Germany). RNA yield and purity were assessed using a DS‐11 FX+ Spectrophotometer (DeNovix, Wilmington, Delaware, USA; Tetreault et al., 2025). Between three and five biological replicates were used for every RNA characterization.

RNA fragmentation was characterized by electrophoresis using one of two Bioanalyzers (Agilent, Waldbronn, Germany) with Agilent RNA 6000 Pico chips and the Plant RNA Pico assay (Agilent 2100 Expert software version B.0208.SI648 R3). Evaluation of RNA quality was assessed using Agilent's “RIN” (RNA Integrity Number) calculation, which is a proprietary formula based on the ratio of peak size for the 18 s and 25 s RNAs as well as characteristics of the electropherogram in the faster‐eluting regions (i.e., those with shorter molecules; Schroeder et al., 2006).

### Aging/longevity assessments and statistical comparisons

Lower germination proportions or RIN values of stored cohorts in comparison to recently harvested cohorts were considered evidence that seeds had aged (i.e., ∆germ > 0 and ∆RIN > 0). These comparisons were confounded when the initial quality of the seeds was low, due to absent or damaged embryos, or when dormancy could not be broken. Expressing the denominator of germination proportion in terms of filled seeds (i.e., number of seeds sown minus number of empty seeds) gave a reasonable correction for empty seeds in the germination assay and was consistent with RIN assessments, which mostly filtered out empty seeds from RNA extractions (Tetreault et al., 2025). We also noted less dormancy in some stored seeds than in their recently harvested counterparts (Walters et al., 2005a), which could lead to artifactually lower ∆germ for some species. Artifactually lower or higher ∆germ and ∆RIN might also arise from disproportionate levels of damaged embryos in either cohort. Anomalies in viability measurements were often detected using TZ assays. Nevertheless, a TZ correction was not used here because TZ assays were not conducted for all seeds pairs.

We calculated descriptive statistics, compared means, and tested for correlation using Excel's Analysis Toolpak for statistical functions. The overall mean (±SD) RIN values for the recently harvested and stored cohorts were 7.6 ± 1.3 and 6.8 ± 1.7, respectively (Tetreault et al., 2025). The overall mean (±SD) germination proportions for the recently harvested and stored cohorts were 0.67 ± 0.29 and 0.50 ± 0.36, respectively, without correcting for proportion of empty seeds (Walters et al., 2025). Correcting for the proportion of unfilled seeds increased the average germination proportion and decreased the SD among all samples by ~10% and, importantly, normalized differences in germination proportion between cohorts.

The effects of time can be expressed as a rate (i.e., speed of decline) or as a shelf life (i.e., time the sample stays useful). These two ways of quantifying timed effects are reciprocals of each other, such that fast aging (i.e., large change/time) implies short life span (i.e., short shelf life). Mortality is more often expressed in terms of shelf life, or longevity, because viability loss with time is a nonlinear function and so the slope varies drastically with time. Having multiple sampling intervals increases the reliability of assessing the effects of time, and there was a subset of 12 species for which NLGRP stored additional cohorts or samples from nearby populations for different durations. For this subset, we calculated longevities and aging rates from germination and RIN data, respectively. We fitted germination data to the Avrami equation using a linear regression of log transformed data using Excel's “linest” function and calculating the time for a 50% decline in initial germination (P50; Walters et al., 2005a; Fleming et al., 2019; Tetreault et al., 2024). Aging rate based on changed germination could then be expressed as the reciprocal, P50^−1^. We also regressed RIN vs. storage time data using Excel's “linest” function and used the slope of that regression to characterize aging rate based on changed RIN. Aging rates based on germination and RIN observations were then correlated for this subset of 12 species, again using Excel's “linest” function and calculations for the *F*‐statistic and corresponding *P*‐values.

For most of the species in the study, there were just two time points, which allowed us to assess how much aging was apparent within the storage period for each aging metric by using a comparison of germination or RIN between cohorts (i.e., ∆germ or ∆RIN). Aging indicators based on germination and RIN observations were then correlated for the entire group of species, again using Excel's “linest” function and calculations for the *F*‐statistic and corresponding *P*‐values.

The low correlation coefficient, but high statistical significance, in the ∆germ × ∆RIN correlation indicated germination and RIN, and both detected aging, but differently—perhaps on different time scales. One goal of this study was to rank species in terms of their seed longevities, and rankings based solely on either ∆germ or ∆RIN would differ. For this reason, we developed a longevity index (LI) to provide a consensus score for how long seeds are expected to survive. The LI combines evidence of deterioration from these two response variables in equal importance in order to rank longevity among species:

(1) $$\text{Evidence}\;\text{of}\;\mathrm{aging}=|\Delta \text{germ}|\times |\Delta \text{RIN}|/{\text{RIN}}_{\text{initial}}100,$$

(2) LI=storagetime÷evidenceofaging

in which ∆germ and ∆RIN are the differences of germination proportions and RIN between the recently harvested and stored cohorts, and RIN_initial_ is assumed to be close to RIN values for the recently harvested cohort. Absolute values for comparisons among cohorts keep minute differences positive and flag which recently harvested seeds might be damaged or highly dormant. Note that the numerator of LI is time and the denominator is observed change. Since the storage time is fairly similar among the stored samples, the ∆germ and ∆RIN parameters in the denominator drive the variation of LI among species. Hence, smaller detected change implies longer life spans, and so species with the highest LI are expected to produce the longest‐lived seeds.

## RESULTS

### Viability vs. RIN

**Figure 1:**
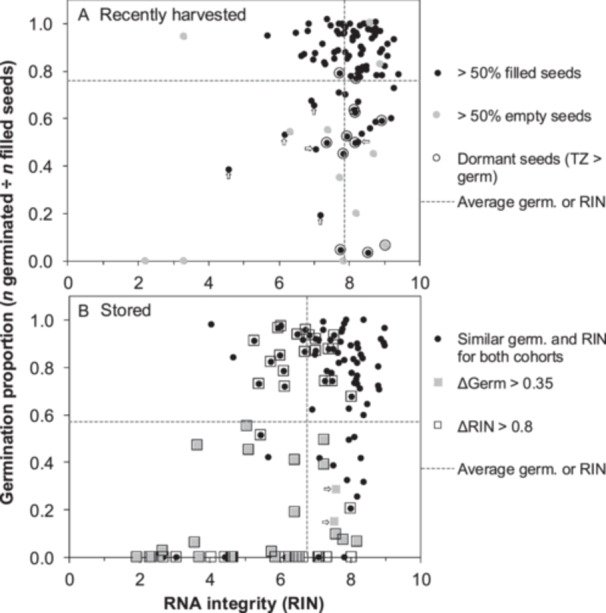
Relationship between germination and RNA integrity of (A) recently harvested and (B) stored cohorts used in this study. There are 104 and 108 populations represented in A and B, each representing a single population of a species. Germination proportion is based on the number of filled seeds (i.e., embryos present; these quality assessments are listed for each species pair in Table 1). Dashed lines represent average germination proportion (horizontal) and RIN (vertical) calculated within each cohort. The symbols differ in the two graphs. For the recently harvested cohort (A), various factors affecting seed germination are plotted to show that germination below the average arises from dormancy (points are encircled if TZ staining suggests that viability > germination proportions) or seed fill (points are black or gray if >50% or <50% of the seeds have embryos, respectively). Arrows point to seed samples that had shriveled embryos, which may explain lower germination as well (e.g., *Hesperocyparis forbesii*, *Hibiscus dasycalyx*, *Polemonium occidentale* ssp. *lacustre*, *Sidalcea nelsoniana*, *Sisyrinchium sarmentosum*, and *Ziziphus celata*). Efforts were made to remove seeds with no or shriveled embryos before RNA extraction if healthy embryos were apparent. The same factors affect germination in the stored cohorts (Figure 1B), but in different species; for example, arrows point out samples of *Deinandra mohavensis* and *Echinocactus horizonthalonius* var. *nicholii* that had shriveled embryos. In B, values for germination and RIN are similar between cohorts for several of the pairs (black circles). Large differences between cohort pairs, indicating substantial aging or low initial quality of the stored cohort, are indicated for germination (gray squares) or RIN (boxed‐in points), such that a stored sample with both lower germination and RIN, compared with its recently harvested counterpart, is represented by a boxed‐in gray square.

Germination behavior for this set of seeds was previously reported using the acronym D.E.A.D. (dormant, empty, aged, and damaged; Walters et al., 2025). Most of the recently harvested samples exhibited some form of seed dormancy that was eventually broken by stratification, scarification, or application of GA_3_. Average germination proportion among species was 0.67 ± 0.29, and 67% of the seeds germinated within 74 ± 88 d (data not shown; Walters et al., 2025; here and below, results are presented ± SD). When corrected for the proportion of unfilled seeds (0.18 ± 0.24), the germination proportion in the recently harvested cohorts still ranged from 0 to 1, with an overall average of 0.76 ± 0.26 (Table 1; Figure 1A, dashed horizontal line). Some seeds stained positively with TZ at higher proportions than germination, suggesting that dormancy was not broken using our procedures (encircled points in Figure 1A). For example, though germination proportion was <0.1 for seeds of *Actaea arizonica*, *Aletes humilis*, and *Oxypolis canbyi* (encircled points near *x*‐axis in Figure 1A), the proportion of positive TZ assessments ranged from 0.42 to 0.71 (data not shown; Walters et al., 2025). Low or no germination was observed in samples having >94% unfilled seeds (e.g., *Abies fraseri*, *Packera franciscana*, and *Remya kauaiensis*; indicated by gray circles on *x*‐axis in Figure 1A). Some samples from the recently harvested cohort had 20%–40% inviable, shriveled embryos (e.g., *Hesperocyparis forbesii*, *Hibiscus dasycalyx*, *Polemonium occidentale* ssp. *lacustre*, *Sidalcea nelsoniana*, *Sisyrinchium sarmentosum*, and *Ziziphus celata*; indicated by arrows in Figure 1A), which was detected upon dissection as well as during TZ staining.

**Table 1:** Species and seed sources in this study, including data on seed quality found previously in regard to germination capacity (Walters et al., 2025) and RNA integrity (RIN; Tetreault et al., 2025). Germination proportion, expressed here as number of seeds germinating divided by number of seeds sown, is corrected for unfilled seeds in the figures.

	**Recently harvested**			**Stored**				
**Species**	**Age (yr)**	**Germination proportion** a	**RIN**	**Age (yr)**	**Germination proportion** a	**RIN**	**Longevity index** b	**Longevity rank** b
*Abies fraseri*	1.0	0.00	7.8	18.0	0.00	6.2	0.659	38
*Abronia umbellata* ssp. *breviflora*	1.5	0.88	7.6	32.6	0.91	7.7	14.428	4
*Actaea arizonica*	1.4	0.03	8.5	29.3	0.00	7.8	1.009	30
*Agalinis skinneriana* or *A. densiflora*	1.2	0.86	9.1	25.2	0.90	9.0	4.427	14
*Aletes humilis*	1.5	0.06	9.0	34.5	0.20	8.0	0.196	63
*Amaranthus pumilus*	0.5	1.00	8.4	35.2	0.02	5.8	0.008	90
*Amelanchier nantucketensis*	0.8	0.78	8.9	28.0	0.92	8.9	2.252	21
*Amorpha herbacea* var. *crenulata*	0.5	0.97	6.8	20.7	0.45	5.1	0.012	87
*Amsonia tharpii*	0.8	0.91	7.2	34.5	0.92	7.4	92.145	1
*Anemone patens* var. *multifida*	0.5	0.97	8.7	28.0	0.71	8.3	0.237	54
*Arctostaphylos catalinae*	1.0	0.64	8.1	29.0	0.71	8.8	6.019	10
*Argemone glauca*	1.8	0.96	7.7	27.7	0.91	5.3	0.130	65
*Aster furcatus* or *Eurybia furcata*	0.5	0.82	9.0	23.0	0.60	8.4	0.137	64
*Astragalus albens*	0.8	0.95	8.2	27.0	1.00	8.4	7.540	8
*Astragalus bibullatus*	0.8	0.85	7.0	29.0	0.62	6.9	1.586	25
*Astragalus linifolius* or *A. rafaelensis*	0.8	0.50	7.4	34.5	0.73	8.2	2.016	22
*Astragalus magdalenae* var. *peirsonii*	2.2	1.00	7.8	20.0	0.92	7.0	0.216	57
*Astragalus tegetarioides* c, f				25.0	0.93	7.6		
*Astragalus tyghensis*	1.8	0.97	7.7	23.5	0.78	7.5	0.473	42
*Berberis nevinii* d	1.0	0.96	7.9	33.9	0.87	7.0	0.311	48
*Besseya bullii*	1.8	0.97	8.1	37.5	0.94	6.8	0.645	39
*Bidens torta*	1.5	1.00	8.6	21.0	0.86	6.7	0.054	75
*Boechera parishii* or *Arabis parishii*	2.4	0.88	7.9	32.0	0.96	7.2	0.444	43
*Bromus carinatus* var. *carinatus*	0.5	0.98	7.9	19.0	0.97	6.0	0.366	47
*Calochortus umpquaensis*	2.2	1.00	7.7	30.7	0.97	6.5	0.531	40
*Campanula scabrella*	1.1	0.79	9.4	29.7	0.06	8.2	0.028	84
*Carex comosa*	0.5	0.67	6.9	19.0	0.91	7.1	0.310	49
*Carex oronensis*	0.8	0.86	6.6	32.0	0.82	5.8	0.503	41
*Castela emoryi*	1.3	1.02	7.4	17.3	0.55	5.1	0.008	89
*Castilleja kaibabensis*	1.8	0.97	8.9	34.0	0.50	7.2	0.032	79
*Ceanothus cyaneus*	1.5	0.77	8.2	33.1	0.73	8.1	8.777	7
*Chenopodium oahuense*	1.3	0.55	7.4	25.0	0.52	5.5	0.196	62
*Chrysopsis floridana*	2.0	0.83	8.9	34.5	0.67	8.0	0.211	58
*Cimicifuga elata*	1.5	0.52	7.9	29.0	0.84	8.3	1.269	27
*Cirsium pitcheri*	1.5	0.59	8.9	32.0	0.42	8.3	0.238	53
*Clarkia biloba* ssp. *australis*	0.5	1.00	8.4	33.0	0.95	7.8	0.739	34
*Clematis socialis*	0.8	0.45	7.8	29.7	0.00	4.6	0.009	88
*Clermontia kakeana*	2.4	0.78	8.8	25.7	0.78	6.1	0.952	31
*Cordylanthus maritimus* ssp. *palustris*	2.3	0.80	8.3	33.0	0.74	7.3	0.374	46
*Cyanea angustifolia*	0.8	0.81	8.3	26.0	0.49	8.0	0.218	56
*Cyperus javanicus*	0.5	0.95	5.7	14.7	0.00	2.4	0.001	104
*Dalea foliosa*	1.3	0.99	7.8	22.0	0.96	7.8	9.541	5
*Deinandra increscens* ssp. *villosa*	2.0	0.92	9.1	19.0	0.88	7.4	0.203	60
*Deinandra mohavensis*	0.5	0.67	8.3	19.0	0.29	7.6	0.058	74
*Dicerandra immaculata*	1.8	0.54	6.3	35.0	0.00	2.3	0.004	99
*Dodonaea viscosa*	1.5	0.95	8.2	32.8	0.72	6.1	0.043	77
*Dubautia menziesii*	0.8	0.20	8.2	19.5	0.00	7.1	0.063	73
*Echinacea tennesseensis*	0.8	0.93	8.1	29.0	0.76	8.2	2.373	20
*Echinocactus horizonthalonius* var. *nicholii*	2.6	0.85	7.9	32.5	0.15	7.5	0.085	70
*Erigeron parishii*	2.8	0.90	8.3	32.0	0.88	7.5	1.201	28
*Eriogonum crosbyae* c				38.0	0.00	6.5		
*Eriogonum cusickii*	0.5	0.81	8.5	40.0	0.00	6.3	0.014	86
*Eryngium aristulatum* var. *parishii*	0.8	0.97	8.6	33.3	0.74	7.5	0.099	68
*Eustachys petraea*	0.7	0.87	6.7	15.0	0.47	3.7	0.004	96
*Eutrema penlandii* or *E. edwardsii*	1.8	0.89	8.4	35.5	0.80	8.8	5.843	11
*Gentiana newberryi*	0.5	0.60	9.2	29.0	0.07	7.8	0.031	81
*Geum geniculatum*	1.2	0.94	8.6	33.5	0.00	4.7	0.004	98
*Gilia leptantha* ssp. *leptantha*	1.3	0.62	8.2	19.6	0.39	7.5	0.092	69
*Hedeoma diffusum*	0.8	0.89	8.9	33.5	0.39	7.2	0.028	83
*Helonias bullata*	0.7	0.79	8.3	33.0	0.75	8.2	7.357	9
*Hesperocyparis forbesii* or *Cupressus guadalupensis* var. *forbesii*	1.0	0.53	6.2	26.3	0.42	5.7	0.270	52
*Hibiscus dasycalyx*	1.8	0.66	7.0	29.8	0.99	7.2	0.288	50
*Horkelia hendersonii*	2.3	0.58	8.8	34.3	0.32	8.4	0.283	51
*Hymenoxys texana*	0.5	0.98	8.7	16.0	0.96	6.7	0.231	55
*Kalmiopsis fragrans*	1.6	0.54	8.4	19.2	0.50	8.1	1.912	23
*Leiophyllum buxifolium* or *Kalmia buxifolia*	2.3	0.70	7.9	28.5	0.00	2.4	0.002	102
*Liatris novae‐angliae* c				32.8	0.96	9.0		
*Lilium parryi*	0.5	1.00	8.0	31.5	0.89	8.0	3.930	15
*Linum carteri* var. *carteri*	1.0	0.96	8.2	19.0	0.98	6.0	0.390	45
*Lomatium bradshawii*	0.8	0.87	8.3	32.0	0.82	7.7	0.684	37
*Lupinus westianus* var. *aridorum* e	0.8	0.83	7.3	11.5	0.73	5.4	0.033	78
*Lycium sandwicense*	1.8	1.00	6.9	16.5	0.85	6.0	0.078	71
*Metrosideros polymorpha* var*. polymorpha*	2.5	0.45	8.7	25.3	0.33	7.9	0.201	61
*Muhlenbergia microsperma*	0.6	0.50	8.2	14.0	0.63	8.0	0.440	44
*Nolina brittoniana*	1.8	0.84	7.3	36.5	0.86	7.0	4.618	13
*Ornithostaphylos oppositifolia*	1.0	0.56	8.6	32.0	0.10	7.6	0.052	76
*Osteomeles anthyllidifolia*	1.5	0.79	7.7	22.7	0.41	6.4	0.029	82
*Oxypolis canbyi*	2.1	0.05	7.8	34.5	0.00	4.4	0.101	67
*Packera franciscana* f or *Senecio franciscanus*	1.3	0.00	2.2	33.0	0.25	8.2	1.770	24
*Penstemon clutei*	1.7	0.89	7.7	31.0	0.03	2.7	0.002	101
*Penstemon peckii*	2.4	0.94	8.1	29.0	0.85	8.3	4.665	12
*Penstemon shastensis*	1.3	0.96	7.8	30.0	0.06	3.6	0.003	100
*Phacelia formosula*	0.5	0.96	8.8	34.0	0.90	8.2	0.822	32
*Physaria globosa*	2.4	0.97	7.7	26.5	0.82	8.2	2.457	19
*Physaria obcordata*	2.1	0.82	8.4	36.3	0.19	6.4	0.019	85
*Pinus radiata* c				17.0	0.92	6.6		
*Pityopsis ruthii*	2.1	0.95	8.7	28.5	0.00	5.9	0.006	94
*Plagiobothrys hirtus*	0.8	0.99	8.3	35.0	1.00	7.9	9.224	6
*Polemonium eddyense* or *P. chartaceum*	4.3	0.78	8.0	30.5	0.86	7.6	0.703	35
*Polemonium occidentale* ssp. *lacustre* f	2.1	0.39	4.6	23.5	0.83	7.8	0.689	36
*Polyscias racemosa*	1.8	1.00	8.8	24.0	0.00	6.6	0.007	91
*Ptilimnium nodosum*	1.5	0.35	7.7	35.0	0.00	3.1	0.007	93
*Purshia subintegra*	0.5	0.82	8.5	24.5	0.72	8.8	3.704	17
*Remirea maritima* f	1.3	0.95	3.3	19.3	0.00	2.6	0.007	92
*Remya kauaiensis* f	0.5	0.00	3.3	32.0	0.00	4.6	1.132	29
*Rhus kearneyi* ssp. *kearneyi*	1.6	0.78	8.3	37.4	0.78	7.3	3.878	16
*Sarracenia oreophila*	2.9	0.85	7.6	34.0	0.00	1.9	0.001	103
*Schoenoplectus tabernaemontani*	0.5	0.88	7.0	17.0	0.84	4.7	0.102	66
*Senecio ertterae*	1.7	0.73	9.3	29.3	0.42	7.1	0.031	80
*Sesbania tomentosa*	0.5	0.96	6.5	25.0	0.98	4.1	0.208	59
*Sidalcea nelsoniana*	1.5	0.50	8.3	37.0	0.89	9.0	1.413	26
*Sisyrinchium sarmentosum*	0.5	0.56	7.1	27.5	0.69	8.5	3.000	18
*Solidago plumosa*	0.5	0.93	9.3	18.5	0.78	8.0	0.077	72
*Tephrosia angustissima* var. *corallicola*	1.3	0.99	7.5	23.5	0.99	7.8	76.803	2
*Vaccinium boreale*	1.6	0.87	8.9	27.0	0.83	8.1	0.777	33
*Vaccinium crassifolium* ssp. *sempervirens*	2.8	0.71	7.7	33.0	0.00	3.7	0.004	97
*Warea amplexifolia* d	2.7	0.86	8.0	35.5	0.88	8.2	28.070	3
*Ziziphus celata* e	2.8	0.19	7.2	15.5	0.00	2.7	0.005	95
Average ± SD	1.4 ± 0.8	0.76 ± 0.26	7.9 ± 1.2	27.9 ± 6.7	0.57 ± 0.36	6.8 ± 1.8	0.23 (median)	

^a^ Number of germinating seeds divided by number of filled seeds.

^b^ Longevity index (LI) is based on Equations 1 and 2. If germination proportions for recently harvested and stored cohorts were the same, Δgerm was assigned a value of 0.01, rather than 0. Anticipated survival time was ranked using the LI, with lowest rank indicating longest‐living seeds.

^c^ A recently harvested sample was not available.

^d^ Stored cryogenically (in LN).

^e^ Stored at 5°C.

^f^ Sample not included in ΔGerm × ΔRIN regression due to low quality of the recently harvested cohort (gray square in Figure 5).

RIN values for recently harvested and stored seeds were previously reported by Tetreault et al. (2025), and the average among the recently harvested cohorts was 7.9 ± 1.2 (Table 1; Figure 1A, dashed vertical line). RIN values < 5.5 reliably indicated low‐quality samples that had shriveled embryos or a very low proportion of filled seeds. RIN of dormant seeds (encircled points in Figure 1A) tended to be high and often greater than the overall average RIN, which potentially introduces a new way to distinguish dead and dormant seeds. High RIN values in low‐quality seed lots might be attributed to the dissection step before RNA was extracted, which removed empty and some shriveled seeds when possible. The relatively high variation in RIN values for samples with germination proportions >0.85 (black circle on far right side of Figure 1A) suggests that RIN may not reliably indicate germination potential in fresh seed.

Seeds were harvested nearly three decades ago (average = 28 ± 7 yr) from the same populations as the recently harvested samples and stored at −18°C. A similar plot between germination proportion and RIN for the stored seeds is given in Figure 1B, in which the symbols differ from those used in Figure 1A in order to highlight differences between the two cohorts. When corrected for the proportion of unfilled seeds (average = 0.20 ± 0.24), the germination proportion of stored samples ranged from 0 to 1, with an overall average of 0.57 ± 0.36 (Table 1; Figure 1B, dashed horizontal line), indicating lower germination and greater variation among stored samples than among recently harvested samples (*t*‐test of means using paired samples, *t* = 6.42, df = 104, *P* << 0.01). Also, the coefficient of variation (CV = SD ÷ mean) was larger for stored samples (0.36/0.57 = 0.64) than for recently harvested samples (0.25/0.78 = 0.34). Considerable differences in germination proportion (Δgerm > 0.35) between recently harvested and stored cohorts are indicated by gray squares in Figure 1B (27 points). Some of these differences can be attributed to lower initial quality (i.e., more shriveled or damaged seeds) in the stored samples than in recently harvested samples (*Deinandra mohavensis* and *Echinocactus horizonthalonius* var. *nicholii*; indicated by arrows in Figure 1B).

The average RIN among stored samples was 6.8 ± 1.8 (Table 1; Figure 1B, dashed vertical line) and was lower, with more variable RIN values than that of recently harvested samples (*t*‐test of means using paired samples, *t* = 7.0, df = 104, *P* << 0.01). Also, the coefficient of variation (CV = SD ÷ mean) was larger for stored samples (1.8/6.8 = 0.26) than for recently harvested samples (1.2/7.9 = 0.15). The difference in RIN between stored and recently harvested pairs was >0.8 (i.e., ΔRIN > 0.8) in 57 species (boxed‐in symbols in Figure 1B). A total of 24 species pairs exhibited both Δgerm > 0.35 and ΔRIN > 0.8 (boxed‐in gray squares in Figure 1B), and these species are suspected of being vulnerable to rapid aging during storage (e.g., *Eustachys petraea*, *Gentiana newberryi*, *Geum geniculatum*, *Pityopsis ruthii*, *Polyscias racemosa*, *Sarracenia oreophila*, and *Vaccinium crassifolium* ssp. *sempervirens*). Conversely, there were 48 species pairs in which Δgerm < 0.35 and ΔRIN < 0.8 (black circles in Figure 1B), suggesting some candidate species that may survive longer in genebanks (e.g., *Abronia umbellata* ssp. *breviflora*, *Amelanchier nantucketensis*, *Astragalus albens*, *Ceanothus cyaneus*, *Sisyrinchium sarmentosum*, and *Tephrosia angustissima* var. *corallicola*).

There is no apparent correlation between germination and RIN, despite the apparent tendency for both these variables to decline during storage (Figure 1B). RIN values vary widely in stored seeds exhibiting high germination (>0.85; black circle on far right side of Figure 1B), again indicating that low RIN does not imply low germination potential. Germination was slower in some stored samples with high germination proportions (Walters et al., 2025), suggesting that aging had occurred in some stored samples before it was evidenced by mortality. On or near the *x*‐axis of Figure 1B, RIN values for ungerminated seeds also varied broadly, in contrast to recently harvested seeds in which this variation was explained by dormancy or high incidence of unfilled seeds.

### Effects of storage temperature and time on germination and RIN

**Figure 2:**
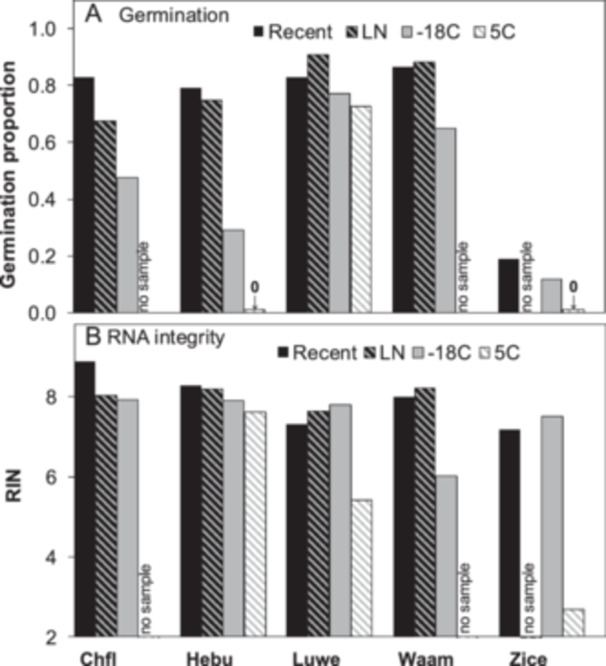
Effect of storage temperature on (A) seed germination and (B) RNA integrity (RIN) in a subset of species having cohorts that were stored either at 5°C (*Lupinus westianus* var. *aridorum* [Luwe] and *Ziziphus celata* [Zice]) or in the vapor phase above liquid nitrogen (LN) (*Chrysopsis floridana* [Chfl], *Helonias bullata* [Hebu], and *Warea amplexifolia* [Waam]). Seeds were stored for 31–34 yr (Chfl, Hebu, Luwe, and Waam) or 12–14 yr (Zice). Samples of the same age and species were collected from nearby populations and stored at −18°C (and additionally at LN for Luwe only) and were tested for germination and RIN to account for differences in storage temperature when comparing longevity among species. Germination proportion is based on the number of filled seeds (i.e., embryos present).

There were six samples of seeds that were stored for ~33 yr under either refrigerated (5°C) or cryogenic (around −180°C) conditions, as well as a sample of *Ziziphus celata* that was stored for 12 yr at 5°C. To account for the effects of storage temperature, we sought seed samples of the same species that were collected from nearby populations and stored for comparable times at −18°C. Germination and RIN were similar or lower in samples stored at −18°C, compared with recently harvested counterparts, consistent with data presented in Figure 1B (Figure 2). There were no detectable differences in germination or RIN in cryogenically stored seeds compared with the recently harvested ones, and seeds stored at 5°C exhibited profoundly lower quality; an analysis of variance showed between‐group effects (*P* < 0.01) on RIN values for all species except *Chrysopsis floridana* (Chfl). The temperature effects on germination proportion (Figure 2A) are well expected, being the reason that genebanks converted to freezer storage ~50 yr ago and that some genebanks explored even lower storage temperatures requiring liquid nitrogen. New data using RIN (Figure 2B) show similar patterns as germination, possibly linking these two types of assays.

**Figure 3:**
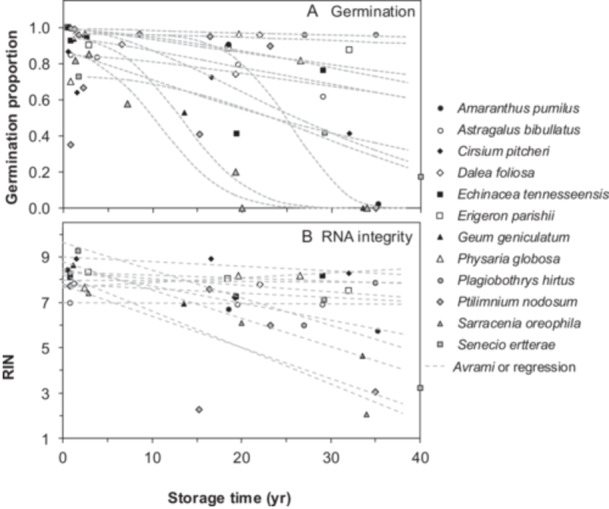
Effect of storage time on (A) seed germination and (B) RNA integrity (RIN) in a subset of species that had additional representatives stored for intermediate durations. These added time treatments come from seeds that were collected from nearby populations in 2005–2010 and stored at −18°C. Germination proportion is based on the number of filled seeds (i.e., embryos present). Three or more data points allowed us to draw deterioration time courses and calculate longevity and aging rate parameters. Dashed curves in A represent data fit to a curvilinear model (the Avrami equation) and show calculations of P50 (time for germination proportion to decline by 50%). P50 values for the fastest‐aging seeds, *Sarracenia oreophila* and *Geum geniculatum*, are 11 yr and 14 yr, respectively. P50 for the longest‐lived seeds, *Plagiobothrys hirtus* and *Physaria globosa*, are extrapolated with large uncertainty to be 559 yr and 191 yr, respectively. In B, dashed lines are linear regressions of RIN vs. storage time using individual biological replicates for each of the three time points (i.e., 12–27 points were used in the regression, but the average RIN value for each point is plotted). RIN correlates with time (*P* < 0.03) for *Amaranthus pumilus*, *Cirsium pitcheri*, *Erigeron parishii*, *Geum geniculatum*, *Ptilimnium nodosum*, *Sarracenia oreophila*, and *Senecio ertterae* (*r* ^2^ range: 0.36–0.89; *n* range: 12–27; slope less than −0.02 RIN yr^−1^). RIN did not correlate with time (*P* > 0.05) for samples of *Astragalus bibullatus*, *Dalea foliosa*, *Echinacea tennesseensis*, *Physaria globosa*, and *Plagiobothrys hirtus* (*n* range: 12–19; slope greater than −0.02 RIN yr^−1^).

Seeds of 12 species were also harvested from nearby populations between 2004 and 2012 and stored at −18°C. Initial germination data from 2004 to 2012 and recent tests of germination and RIN provided seed quality estimates at intermediate storage times (13–20 yr), which allowed us to construct deterioration time courses and calculate aging rates based on declining germination proportion or RIN (Figure 3). Longevity, calculated as P50 (i.e., the time for initial germination to decline by 50%), ranged from 11 yr (*Sarracenia oreophila*) to 559 yr (*Plagiobothrys hirtus*), with a median P50 of 43 yr (Figure 3A). Aging rate was calculated as the reciprocal of P50 (P50^−1^) to facilitate comparisons with rate of RIN decline (Figure 3B). Values of P50^−1^ for the 12 species ranged from 0.65 d^−1^ (slow aging) to 33 d^−1^ (rapid aging) (median = 9.2 d^−1^), suggesting that aging rates among these species differ by nearly two orders of magnitude.

RIN values for each replicate sample (four to ten replicates for three or four time treatments) were regressed against storage time (data in Figure 3B plot average RIN values). Significant effects of time were noted for *Amaranthus pumilus*, *Cirsium pitcheri*, *Erigeron parishii*, *Geum geniculatum*, *Ptilimnium nodosum*, *Sarracenia oreophila*, and *Senecio ertterae* (*F* range: 171.0–9.5; df range: 11–27; *P* < 0.01; slope less than −0.02 RIN yr^−1^; *r* ^2^ range: 0.36–0.89). RIN values did not correlate with time for samples of *Astragalus bibullatus*, *Dalea foliosa*, *Echinacea tennesseensis*, *Physaria globosa*, and *Plagiobothrys hirtus* (*F* range: 8.6–0.1; df range: 10–22; *P* > 0.02; slope greater than −0.02 RIN yr^−1^; *r* ^2^ range: 0.01–0.47); these species are represented by black circles in the upper right quadrant of Figure 1B. A decline in RIN of about −0.02 yr^−1^ separates the species that did and did not show significant correlations with time, which amounts to a total change in RIN of 0.56 over 28 yr; this estimate is consistent with previous estimates of the statistical power of detecting RIN change (Tetreault et al., 2023). Overall, average values of slopes in the RIN vs. time regression analyses for the groups that did and did not correlate with time were −0.073 and −0.008 RIN yr^−1^, respectively.

**Figure 4:**
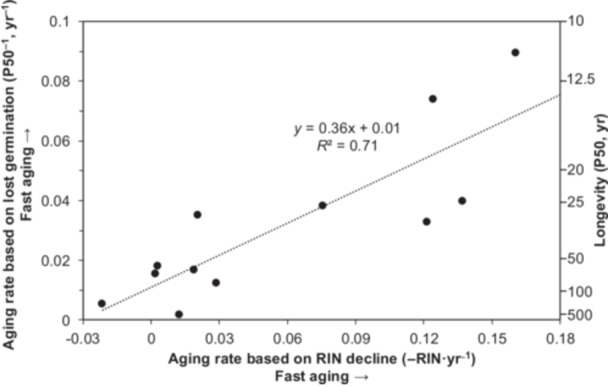
Relationship between aging rates estimated by rate of RIN decline and by lost viability for the 12 species studied in Figure 3. Longevity (right axis) was transformed to aging rate (left axis) using the reciprocal of calculated P50, which also reduces uncertainty when extrapolating viability models. The correlation is significant (*F* = 24.9, df = 10, *P* < 0.01).

The two measures of aging rate calculated from declining germination proportion (P50^−1^; Figure 3A) and RIN (slope of RIN vs. time regression; Figure 3B) were regressed for the 12 species having data for three or more time points (Figure 4). The correlation is significant (*F* = 24.9, df = 10, *P* < 0.01), suggesting that RIN decline may be a suitable method for predicting seed longevity. The secondary *y*‐axis in Figure 4 marks the P50 value corresponding to P50^−1^, which was used in the correlation. This relationship shows that a shelf life of ~15 yr is expected if RIN declines at a rate of 0.15 yr^−1^. By contrast, we expect a shelf life near 68 yr if RIN declines at a rate of 0.01 yr^−1^.

### Do differences in germination and RIN correlate?

**Figure 5:**
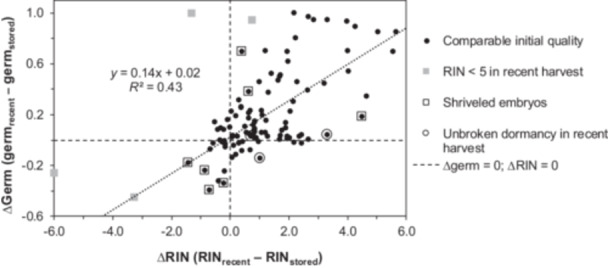
Relationship between aging detected by lower germination and RIN between cohort pairs (see Table 1). The points are calculated as the difference in germination (∆germ) and RNA integrity (∆RIN) between recently harvested and stored seeds. Symbols reflect differences in seed quality after correction for empty seeds (Figure 1A): relatively high germination capacity of the recently harvested cohort (black circles), unbalanced proportions of shriveled embryos (boxed‐in points), or high retention of dormancy. The two methods used to detect aging correlate (*F* = 77.8, df = 102, *P* << 0.01). Samples were excluded from the regression if RIN < 5 in the recently harvested sample (gray squares, as in Figure 1A).

Given the promising correlation we found between rates of declining germination proportion and RIN in samples from 12 species (Figure 4), we tested whether the differences in germination proportion (∆germ) and RIN (∆RIN) were correlated for paired samples that were freshly harvested and stored for 28 ± 7 yr. We excluded four pairs for which the seed quality of the initial sample was aberrantly low (RIN < 5; gray squares in Figure 5 and Figure 1A). The correlation is significant (*F* = 77.8, df = 102, *P* << 0.01), though there is large residual error indicated by a low *r* ^2^ value (0.43; Figure 5), suggesting numerous other contributing factors. Despite one‐fourth of the species pairs not showing changed germination or RIN, there is sufficient variation in the bulk of the data set to study correlations between ∆germ and ∆RIN, as these values ranged from −0.39 to 1.00 (average = 0.20 ± 0.33) and from −1.43 to 5.65 (average = 1.1 ± 1.7), respectively. Differences in initial seed quality between the seed lot pairs will confound the regression. We made corrections for differences caused by empty seeds by expressing germination proportion on the basis of number of filled seeds. We noted the few samples with high amounts of shriveled embryos in either cohort using boxed‐in symbols, but still included these points in regression calculations (Figure 5). We also indicated points in which dormancy was retained in the recently harvested sample (encircled points in Figure 5); low ∆germ values are calculated for species with high dormancy in both cohorts. The cluster of points around the origin represent about one‐fourth of the species that did not show differences between recently harvested and stored seed lots (also shown as black points in Figure 1B). Differences in the timing with which RIN and germination declined may explain some of the variation in the regression. For example, RIN may be low but germination high in recently harvested cohorts of rapidly aging seeds (Figure 1A, upper left quadrant), giving the appearance of low ∆RIN and high ∆germ (e.g., Figure 5; *Cyperus javanicus*, ∆RIN = 3.3, ∆germ = 0.95). Alternatively, a high ∆RIN and low ∆germ may be observed in stored seeds approaching an aging threshold that portends rapid mortality (e.g., Figure 5; *Clermontia kakeana*, ∆RIN = 2.7, ∆germ < 0.01).

**Figure 6:**
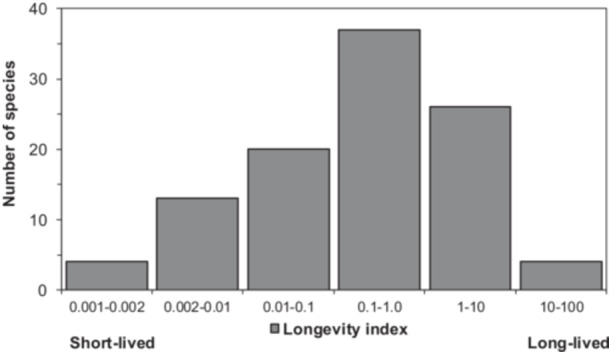
Distribution of 104 species along a relative scale of seed longevity based on aging detected among cohort pairs collected 28 ± 7 yr apart. Relative longevity was calculated by a longevity index (LI; Equation 2) that uses differences in both germination (∆germ) and RNA integrity (∆RIN) (as represented by the two axes in Figure 5). This factor (Equation 1) is multiplied by the storage time that separates the two cohorts (Equation 2). The median value for the LI is 0.28. Seeds from the stored samples that are identified as quite short‐lived (LI < 0.002), that did not germinate in recent assays (germination proportion ≤ 0.03), and that had highly degraded RNA (RIN ≤ 2.65) are from *Cyperus javanicus*, *Leiophyllum buxifolium*, *Penstemon clutei*, and *Sarracenia oreophila*. Seeds from the stored samples that are identified as long‐lived (LI > 10), that exhibited germination proportions >0.88, and having RIN values > 7.4 are *Abronia umbellata* ssp. *breviflora*, *Amsonia tharpii*, *Tephrosia angustissima* var. *corallicola*, and *Warea amplexifolia* (*W. amplexifolia*, however, was stored cryogenically). Seeds of *Liatris novae‐angliae* may also be long‐lived, based on germination proportion and RIN of stored seeds (0.96 and 9.0, respectively); however, a recently harvested cohort for this species was not available. LI values and rankings for longevity for all species in the study are listed in Table 1.

Because species varied in how they expressed the two aging signals, we combined the ∆germ and ∆RIN signals to assess how much deterioration was detected and to rank species accordingly (Equation 1). Species that showed both high ∆germ and ∆RIN were deemed to age quickly relative to species that showed low ∆germ and ∆RIN. To rank species by longevity rather than aging rate, we divided the storage time by the composite aging signal, resulting in a longevity index (Equation 2; median LI = 0.25). The >100 species in this study are binned into six longevity categories (Figure 6; Table 1 provides ranking). Very short‐lived (LI < 0.01) species include *Cyperus javanicus*, *Eustachys petraea*, *Geum geniculatum*, *Leiophyllum buxifolium*, *Penstemon shastensis*, *Pityopsis ruthii*, and *Sarracenia oreophila*; germination proportions in these stored cohort were near zero except for *Eustachys petraea* where germination proportion = 0.47. RIN for apparently fast aging seeds was <6, except for *Polyscias racemosa* (where RIN = 6.6). By contrast, apparently long‐lived seeds (LI > 10) included *Abronia umbellata* ssp. *breviflora*, *Amsonia tharpii*, *Dalea foliosa*, and *Tephrosia angustissima* var. *corallicola*; the stored cohorts of these species all exhibited germination proportions >0.90 and RIN > 7.4. Other long‐lived seeds are ranked in the top 25 (Table 1, rightmost column) and include *Astragalus albens*, *Ceanothus cyaneus*, *Eutrema penlandii*, *Nolina brittoniana*, *Penstemon peckii*, *Plagiobothrys hirtus*, and *Sisyrinchium sarmentosum*. Germination proportions were >0.70 and RIN values were >6 in the stored cohorts of these purportedly long‐lived seeds.

## DISCUSSION

This study explored seed responses to genebanking among wild species native to the United States using a set of paired seed samples from >100 species collected for conservation purposes and stored in genebanks (FAO, 2014; CPC, 2019). One cohort of the pair was collected between 1983 and 2010 (average storage time: 28 ± 7 yr), and the other cohort was collected from the same populations in 2021–2024. Two methods to assess aging were used, and average germination proportion and RIN were both significantly lower in the stored cohort than in the recently harvested cohort (Tetreault et al., 2025; Walters et al., 2025). This is reflected by Δgerm ranging from 0.14 to 0.27 (95% CI, *α* = 0.05, average = 0.20 ± 0.33, median = 0.09) and ΔRIN ranging from 0.80 to 1.44 (95% CI, *α* = 0.05, average = 1.1 ± 1.7, median = 0.85). The present study differs from preceding studies in this series that explored factors of wild seeds that might influence the reliability and implementation of assays to detect seed deterioration during storage. Here, we hypothesized that the diversity of species and their habitats would reveal a wide range of seed responses to genebanking, and we sought to identify species that were either short‐ or long‐lived. Our approach fundamentally differs from other surveys of seed longevity from regional flora because it is based on decades‐long storage under actual genebanking conditions, rather than inferring applicability to genebanks from data collected on seeds that died in a matter of weeks under warm, humid conditions.

Germination assays are the “gold standard” used to test seed quality and to detect change during seed storage (FAO, 2014; AOSA, 2018; CPC, 2019). The test results depend on using temperature and moisture conditions that can elicit germination—conditions that, along with the speed at which seeds germinate, can vary widely among species, especially among wild species (Walters et al., 2025). Managing this kind of diversity is a genebanking goal that must be balanced with the need for calibrated and standardized approaches to ensure quality and affordability of operations. TZ testing, which assesses viability based on vital staining patterns, provides results in 1–2 d using a more standardized approach that appears to be less affected by seed dormancy. TZ tests do not reveal whether a normal seedling will develop—a point that is essential for assessing survival and overall seed health; moreover, knowledge about germination requirements enhances the utility of stored germplasm. However, the primary goal for the genebank is to detect a change in quality of the stored sample before it loses utility. Both germination and TZ tests present limitations in accomplishing this goal because they are initially insensitive to changes with time. These tests produce binary data (viable/inviable) that are quantified by proportions, which require relatively large sample sizes to detect minor differences (Tetreault et al., 2023). Proportion data follow sigmoidal decay curves, which tend to have shallow slopes initially (Figure 3A). TZ assays may be even less sensitive to time than germination assays, and this lower sensitivity is often compounded by the use of small sample sizes (Hay and Probert, 2013; Fleming et al., 2017, 2019; DeVitis et al., 2020; Walters et al., 2004, 2005a, 2005b, 2010, 2020; Nadarajan et al., 2023; Tetreault et al., 2023, 2024). Though aging is difficult to detect initially, it culminates in a threshold beyond which seeds die rapidly (Figure 3A) and the sample loses its utility. The genebanking community, therefore, focuses on anticipating when this threshold occurs and defines this time as the shelf life or longevity of the seed lot. Often, the timing of this threshold is discovered after the fact, when a germination test reveals mostly dead seeds.

Greater focus on the initial phase of deterioration time courses may prospectively reveal the longevity of seeds. This will require analyses that are sensitive to change during early storage. Here, we use RIN, which degrades linearly with time throughout storage (Figure 3B) and so changes can be detected well before lost viability is apparent (Fleming et al., 2017, 2019; Walters et al., 2020; Zhao et al., 2020; Saighani et al., 2021; Tetreault et al., 2023, 2024, 2025). RNA extraction and characterization are standardized and are unaffected by seed morphology or germination behavior typical of wild seeds (Tetreault et al., 2025; in Figure 1A, encircled points show high RIN of dormant seeds). Low RIN values (RIN < 6) may indicate poor seed quality. However, RIN and germination proportion do not correlate (Figure 1) because RIN is high in dormant seeds and because germination proportion doesn't change much initially or after all seeds have died, even though RIN declines progressively. Nevertheless, our results show that both germination and RIN change as expected in relation to storage temperature (Figure 2).

As hypothesized, the evidence of aging varied among the species in the study set. About one‐fourth of the species pairs exhibited no evidence of aging (Δgerm < 0.14, ΔRIN < 0.3); another one‐fourth of the species pairs showed substantial evidence of germination decline (Δgerm > 0.35, ΔRIN > 0.8); and about one‐third of the species pairs showed measurable decline in RIN but less change in germination proportion (Figure 5). The significant correlation between Δgerm and ΔRIN (*P* << 0.01) suggests similar effects of storage time; however, the low *r* ^2^ (0.43) indicates substantial unexplained variation in the expression of aging between the two variables. One source of this variation comes from seeds with overall low germination due to dormancy or damaged embryos. For example, the germination proportions for stored seeds of *Ziziphus celata* and *Geum geniculatum* were both zero and the ΔRIN values were comparable (≈4), suggesting that seeds of each species experienced a similar amount of aging. However, germination proportions for recently harvested seeds were very different (0.19 and 0.94, respectively), resulting in relatively low Δgerm for *Z. celata* despite high ΔRIN (one of the outlying boxed‐in points in Figure 5). In a different example, the high Δgerm for seeds from *Echinocactus horizonthalonius* var. *nicholii* (another outlying boxed‐in point in Figure 5) arises from numerous shriveled embryos in the stored cohort; these were removed prior to RNA extraction. We also think that detection of RIN decline *before* substantial mortality contributes to variation in the regression, such as lower RIN in stored seeds that still germinate in high proportions (boxed‐in points in Figure 1B, high ΔRIN points along the abscissa in Figure 5). We may also see variation from the ΔRIN versus Δgerm relationship as Δgerm approaches its maximum value, ~1.0 (i.e., all filled seeds died), but RIN continues to decline. In this case, seeds that age quite rapidly, such as *Sarracenia oreophila* (Δgerm = 0.85, ΔRIN = 5.65 in Figure 5; see also Figure 3), may have larger ΔRIN than seeds that were slower to age (e.g., *Pityopsis ruthii* (Δgerm = 0.95, ΔRIN = 2.84; Figure 5). We are also exploring a few seed species that appear to be highly resistant to RIN change even after many years of storage (e.g., *Helonias bullata* stored at 5°C [Figure 2B] and papaya and elm seeds; C. Walters, personal observation). Variation of initial seed quality among the two cohorts affects uncertainty of the calculated Δgerm and ΔRIN values and could contribute to some of the negative values observed. Monitoring the same sample over the storage period may provide more robust detection of change but may also deplete the sample.

The rate at which seeds aged can be more explicitly expressed by dividing Δgerm or ΔRIN by the storage time. When rates are constant throughout storage, as we assume with RIN decline (Figure 3B), we can predict when aging will be detectable in a stored seed lot. For example, an average ∆RIN of 1.1 divided by an average storage time of 28 yr leads to an aging rate of 0.039 RIN yr^−1^ (∆RIN ÷ ∆yr = 1.1 ÷ 28). A statistical power assessment concluded that ∆RIN ~ 0.5 was detectable (Tetreault et al., 2023), suggesting that measurable aging might be detected by the RIN assay within 11 yr, well *before* mortality can be detected, which is likely to be ≥28 yr (avg. ∆germ in this study was 0.2). Estimates of aging rate can provide insights on how frequently stored seeds should be monitored for aging and which assays will be most informative. At the current standard of 15 yr (FAO, 2014), a viability monitoring assay may provide little new information, but an RIN assay could distinguish long‐lived (∆RIN < 0.5) from short‐lived (∆RIN > 1) seeds and flag seeds that are on the brink of the mortality threshold (∆RIN > 1.5).

Longevity is a time term and thus is correlated with the reciprocal of aging rate and is often expressed by P50 (the time for germination proportion to decline by 50%). In this study, median P50 is likely to be longer than the storage duration of 28 yr because germination proportion was >0.5 for most of the species in the stored cohort (Figure 1B). Median P50 was 43 yr in the subset of 12 species with more than two time points (Figure 3A) in which average Δgerm = 0.36 and ΔRIN = 1.7 (i.e., larger differences than calculations using the entire set of 104 species). Therefore, estimates of longevity presented here are consistent with a median P50 of 54 yr measured for genebanked crop seeds (Walters et al., 2005a) and provide baseline information about how long genebanked seeds of U.S. wild species survive (Harrison et al., 2023).

Preferably, longevity is expressed in concrete terms, such as years; however, a reliable estimate cannot be calculated with two time points because slopes of viability time courses vary throughout the storage period (e.g., Figure 3A). Even with several time points, calculation of P50 for long‐lived seeds can be unreliable because extrapolated models tend toward infinity when viability changes are small. For example, calculated P50 values for *Plagiobothrys hirtus* and *Physaria globosa* are ~200 yr or more (Figure 3A). Uncertainty from extrapolation can be minimized by expressing longevity as an aging rate (the reciprocal) because that value tends toward a limit of zero for long‐lived seeds. A future goal seeks to correct for extrapolation error using the ΔRIN term. Future analyses will address whether there is a specific amount of RNA degradation that signals the onset of seed mortality. Similarly, other markers of early aging (e.g., intracellular redox, volatile emission, and lipid properties) may also help forecast imminent viability loss. We have introduced the concept of a longevity index (LI) in order to integrate these various markers of early aging to increase the reliability of predicting when mortality occurs in stored seeds (Figure 6). In its current form, the index ranges from <0.01 (fast‐aging seeds) to 100 (slow‐aging seeds) and about one‐third of the species show the central tendency of LI between 0.1 and 1 (median LI = 0.28), which roughly translates to ∆germ of 0.13 and ∆RIN of 0.35 for an average storage time of 28 yr. We anticipate future versions of LI to be informed by how much overall change has occurred, which presumably will serve as “mortality mile markers.” The LI allows us to rank species by how much aging was detected during the storage period (Figure 6). For perspective, *Cirsium pitcherii* is near the median, with LI = 0.24, ∆germ = 0.17, ∆RIN = 0.64, and P50 = 29 yr (p50^−1^ = 12.8; Figure 4), and is ranked 54/105 for longevity among the species. Seeds of *Geum geniculatum* are among the fastest‐aging seeds (ranked 101/105 for longevity, LI = 0.004, Δgerm = 0.94, ΔRIN = 3.98, P50 = 14 yr; Figure 4). *Plagiobothrys hirtus* produces one of the longest‐living seeds (ranked 6/105 for longevity, LI = 9.2, Δgerm = −0.01, ΔRIN = 0.43, P50 = 560 yr, calculated by extrapolating a viability model; Figure 4).

This work aimed to identify seeds with unusually short or long keeping quality. We assumed that the seed lots selected for the study are representative of the species and so the variation we observed is a species‐level trait, which will be considered in the context of phylogeny, habitat, growth habit, and morphological traits (K. D. Heineman et al., unpublished data). Extremely rapid aging in some species (e.g., *Cyperus javanicus*, *Geum geniculatum*, and *Sarracenia oreophila*) is both intriguing and alarming, though it reflects storage physiologies known for pollen, fern spores, and other seeds such as *Salix* (Hoekstra, 2005; Walters et al., 2005b; Ballesteros and Pence, 2019; Ballesteros et al., 2019, 2020). Cryogenic storage may provide an alternative storage platform that prolongs the life spans of these aging‐vulnerable seeds.

## CONCLUSIONS

Genebanking maintained seed germination potential in nearly three‐quarters of seed samples from 108 rare or endangered plant species represented in this study. Overall, seeds in this study are expected to survive >43 yr under genebanking conditions, but there is a small group of seeds that were prone to faster aging. Our ~28 yr monitoring tests used two different methods to assess changed seed quality. Measurements using germination behavior were more variable and less reliable. By contrast, RIN assays provided a standard method to assess the progress of aging that was highly sensitive. Our study suggests that seed longevity can be predicted within a single monitoring interval by the rate of RIN decline. Therefore, RIN assessments can be a promising tool to estimate seed lot “expiration dates” and to more effectively schedule viability monitoring frequencies or early seed replenishment.

## AUTHOR CONTRIBUTIONS

C.W. was co‐PI on the IMLS grant, supervised data collection, analyzed the data, and wrote the manuscript. K.D.H. was PI on the IMLS grant, contributed insights on analyses, helped focus the main points of the study, and contributed to the manuscript. L.H. coordinated laboratory activities, supervised collection of germination data, and contributed to the manuscript. H.T. supervised collection of RIN data during initial phases and contributed to the manuscript. Z.Z. provided technical support for RNA analyses during initial phases and approved the manuscript. S.I. provided technical support for germination and TZ data during initial phases. P.T. provided technical support during later phases that added and confirmed RNA and germ data and approved the manuscript. J.M. was co‐PI on the IMLS grant, provided leadership for ARS and CPC interactions, helped focus the main points of the study, and contributed to the manuscript.

## Supporting information


**Appendix S1.** Information about the source of the seed materials used in this study.
**Table S1.** Institutions from the Center for Plant Conservation that provided botanical expertise and collected the seeds used in this study.
**Table S2.** Species included in this study, and their abbreviations, harvest years for stored and recently harvested cohorts and the institution responsible for identifying the plants and processing the seeds, and in many cases genebanking the accession at −18°C. A key to the institutions is provided in Appendix S1, Table S1.

## Data Availability

Table 1 lists germination proportions (corrected for empty seeds) and RIN for recently harvested and stored cohorts of each species pair and graphs are used to show correlation results. Botanical information about the species included in this study including harvest years, institutions responsible for plant identification and seed preparation and germination behavior were provided previously (Walters et al., 2025). The reliability of RIN data and the effects of factors such as wild seed traits and storage were published previously (Tetreault et al., 2025). Curatorial data for the accessions in this study are available by contacting the CPC National Office (info@saveplants.org).
