# Supplementary material for: Aging and longevity in decades‐old genebanked seeds from U.S. endangered plant species: Assessments using survival and RNA integrity assays
Source: Am J Bot. 2026 Feb 24;113(3):e70169. doi: 10.1002/ajb2.70169 (PMC13003721; doi:10.1002/ajb2.70169)
Supplement: Supplementary file 1 — Appendix S1. Information about the source of the seed materials used in this study. Table S1. Institutions from the Center for Plant Conservation that provided botanical expertise and collected the seeds used in this study. Table S2. Species included in this study, and their abbreviations, harvest years for stored and recently harvested cohorts and the institution responsible for identifying the plants and processing the seeds, and in many cases genebanking the accession at −18°C. A key to the institutions is provided in Appendix S1, Table S1. [file AJB2-113-e70169-s001.docx]

**Appendix S1.** **Information about the source of the seed materials used in this study**

| Table S1. Institutions from the Center for Plant Conservation that provided botanical expertise and collected the seeds used in this study | | |
| --- | --- | --- |
| CPC Institution | Code | Location |
| Atlanta Botanical Garden | ABG | Atlanta, Georgia, USA |
| Rae Selling Berry Seed Bank and Conservation Program | BERR | Portland, Oregon, USA |
| Bok Tower Gardens | BOK | Lake Wales, Florida, USA |
| California Botanic Garden | CalBG | Claremont, California, USA |
| Camcore, North Carolina State University | CAMCORE | Raleigh, North Carolina, USA |
| Chicago Botanic Garden | CBG | Glencoe, Illinois, USA |
| California Native Plant Society | CNPS | Sacremento, California, USA |
| Denver Botanic Gardens | DBG | Denver, Coloroado, USA |
| Desert Botanical Garden | DES | Phoenix, Arizona, USA |
| The Arboretum at Flagstaff | FLAG | Flagstaff, Arizona, USA |
| Fairchild Tropical Botanic Garden | FTBG | Coral Gables, Florida, USA |
| Harold L. Lyon Arboretum | HLA | Honolulu, Hawaii, USA |
| Institute for Applied Ecology | IAE | Corvallis, Oregon, USA |
| Missouri Botanical Garden | MBG | St. Louis, Missouri USA |
| Mercer Arboretum and Botanic Gardens | MERC | Humble, Texas, USA |
| North Carolina Botanical Garden | NCBG | Chapel Hill, North Carolina, USA |
| National Laboratory for Genetic Resources Preservation | NLGRP | Fort Collins, Colorado, USA |
| Native Plant Trust | NPT | Wayland, Massachusetts, USA |
| National Tropical Botanical Garden | NTBG | Kalaheo, Hawaii, USA |
| Santa Barbara Botanic Garden | SBBG | Santa Barbara, California, USA |
| San Diego Zoo Wildlife Alliance | SDZWA | San Diego, Califronia, USA |
| University of Minnesota Landscape Arboretum | UMLA | Minneapolis, Minnesota, USA |
| University of Washington Botanic Gardens | UWBG | Seattle, Washington, USA |

| Table S2. Species included in this study, and their abbreviations, harvest years for stored and recently harvested cohorts and the institution responsible for identifying the plants and processing the seeds, and in many cases genebanking the accession at -18^o^C. A key to the institutions is provided in Appendix S1, Table S1. | | | | | | |
| --- | --- | --- | --- | --- | --- | --- |
| Taxon and synonym | species code | Botanical family | donating institution (recent harvest) | harvest year | donating institution (stored seed) | harvest year |
| Abies fraseri | Abfr | Pinaceae | CAMCORE | 2023 | CAMCORE | 2006 |
| Abronia umbellata var. breviflora | Abum | Nyctaginaceae | IAE | 2021 | BERR | 1990 |
| Actaea arizonica | Acar | Ranunculaceae | FLAG | 2022 | FLAG | 1994 |
| Agalinis densiflora | Agde | Orobanchaceae | CBG | 2021 | CBG | 1997 |
| Aletes humilis | Alhu | Apiaceae | DBG | 2021 | DBG | 1988 |
| Amaranthus pumilus | Ampu | Amaranthaceae | NCBG | 2022 | NCBG | 1987 |
| Amelanchier nantucketensis | Amna | Rosaceae | NPT | 2023 | NPT | 1993 |
| Amorpha herbacea var. crenulata | Amhe | Fabaceae | FTBG | 2021 | FTBG | 2003 |
| Amsonia tharpii | Amth | Apocynaceae | DES | 2023 | DES | 1989 |
| Anemone patens var. multifida | Anpa | Ranunculaceae | UWBG | 2021 | UWBG | 1993 |
| Arctostaphylos catalinae | Arca | Ericaceae | SBBG | 2023 | CalBG | 1995 |
| Argemone glauca | Argl | Papaveraceae | NTBG | 2022 | NTBG | 1996 |
| Aster furcatus or Eurybia furcata | Asfu | Asteraceae | CBG | 2021 | CBG | 1998 |
| Astragalus albens | Asal | Fabaceae | CalBG | 2021 | CalBG | 1995 |
| Astragalus bibullatus | Asbi | Fabaceae | MBG | 2021 | MBG | 1993 |
| Astragalus linifolius or Astragalus rafaelensis | Asli | Fabaceae | DBG | 2023 | DBG | 1987 |
| Astragalus magdalenae var. peirsonii | Asma | Fabaceae | CalBG | 2021 | CalBG | 2003 |
| Astragalus tegetarioides | Aste | Fabaceae |  |  | BERR | 1999 |
| Astragalus tyghensis | Asty | Fabaceae | BERR | 2022 | BERR | 2000 |
| Berberis nevinii | Bene | Berberidaceae | CalBG | 2021 | CalBG | 1990 |
| Besseya bullii | Bebu | Scrophulariaceae | UMLA | 2022 | CBG | 1986 |
| Bidens torta | Bito | Asteraceae | HLA | 2022 | HLA | 2000 |
| Boechera parishii or Arabis parishii | Bopa | Brassicaceae | CalBG | 2021 | CalBG | 1991 |
| Bromus carinatus var. carinatus | Brca | Poaceae | SDZWA | 2024 | SDZWA | 2005 |
| Calochortus umpquaensis | Caum | Liliaceae | BERR | 2022 | BERR | 1993 |
| Campanula scabrella | Casc | Campanulaceae | CNPS | 2023 | CalBG | 1994 |
| Carex comosa | Caco | Cyperaceae | UWBG | 2022 | UWBG | 2003 |
| Carex oronensis | Caor | Cyperaceae | NPT | 2024 | NPT | 1991 |
| Castela emoryi | Caem | Simaroubaceae | CBG | 2021 | CBG | 2004 |
| Castilleja kaibabensis | Caka | Orobanchaceae | FLAG | 2021 | FLAG | 1989 |
| Ceanothus cyaneus | Cecy | Rhamnaceae | SDZWA | 2021 | CalBG | 1990 |
| Chenopodium oahuense | Choa | Amaranthaceae | NTBG | 2022 | NTBG | 1998 |
| Chrysopsis floridana | Chfl | Asteraceae | BOK | 2021 | BOK | 1989 |
| Cimicifuga elata | Ciel | Ranunculaceae | IAE | 2022 | BERR | 1994 |
| Cirsium pitcheri | Cipi | Asteraceae | CBG | 2021 | CBG | 1991 |
| Clarkia biloba ssp. australis | Clbi | Onagraceae | CNPS | 2023 | CalBG | 1991 |
| Clematis socialis | Clso | Ranunculaceae | ABG | 2021 | NCBG | 1993 |
| Clermontia kakeana | Clka | Campanulaceae | HLA | 2021 | HLA | 1997 |
| Cordylanthus maritimus ssp. palustris | Coma | Orobanchaceae | IAE | 2021 | BERR | 1990 |
| Cyanea angustifolia | Cyan | Campanulaceae | HLA | 2021 | HLA | 1997 |
| Cyperus javanicus | Cyja | Cyperaceae | NTBG | 2022 | NTBG | 2008 |
| Dalea foliosa | Dafo | Fabaceae | MBG | 2022 | MBG | 2000 |
| Deinandra increscens ssp. villosa | Dein | Asteraceae | SBBG | 2021 | SBBG | 2003 |
| Deinandra mohavensis | Demo | Asteraceae | CalBG | 2021 | CalBG | 2002 |
| Dicerandra immaculata | Diim | Lamiaceae | BOK | 2022 | BOK | 1987 |
| Dodonaea viscosa | Dovi | Sapindaceae | NTBG | 2022 | NTBG | 1990 |
| Dubautia menziesii | Dume | Asteraceae | HLA | 2021 | HLA | 2002 |
| Echinacea tennesseensis | Ecte | Asteraceae | MBG | 2021 | MBG | 1994 |
| Echinocactus horizonthalonius var. nicholii | Echo | Cactaceae | DES | 2022 | DES | 1991 |
| Erigeron parishii | Erpa | Asteraceae | CalBG | 2021 | CalBG | 1991 |
| Eriogonum crosbyae | Ercr | Polygonaceae |  |  | BERR | 1983 |
| Eriogonum cusickii | Ercu | Polygonaceae | BERR | 2022 | BERR | 1983 |
| Eryngium aristulatum var. parishii | Erar | Apiaceae | SDZWA | 2022 | CalBG | 1990 |
| Eurybia furcata or Aster furcatus | Eufu | Asteraceae | CBG | 2021 | CBG | 1998 |
| Eustachys p3traea or petraea | Eup3 | Poaceae | FTBG | 2024 | FTBG | 2009 |
| Eutrema penlandii or Eutrema edwardsii | Eupe | Brassicaceae | DBG | 2022 | DBG | 1988 |
| Gentiana newberryi | Gene | Gentianaceae | BERR | 2023 | BERR | 1994 |
| Geum geniculatum | Gege | Rosaceae | NCBG | 2021 | NCBG | 1988 |
| Gilia leptantha ssp. leptantha | Gile | Polemoniaceae | CalBG | 2022 | CalBG | 2003 |
| Hedeoma diffusum | Hedi | Lamiaceae | FLAG | 2021 | FLAG | 1988 |
| Helonias bullata | Hebu | Melanthiaceae | ABG | 2024 | NCBG | 1991 |
| Hesperocyparis forbesii or Cupressus guadalupensis var. forbesii | Hefo | Cupressaceae | SDZWA | 2021 | CalBG | 1995 |
| Hibiscus dasycalyx | Hida | Malvaceae | MERC | 2021 | MERC | 1993 |
| Horkelia hendersonii | Hohe | Rosaceae | BERR | 2021 | BERR | 1989 |
| Hymenoxys texana | Hyte | Asteraceae | MERC | 2021 | MERC | 2005 |
| Kalmiopsis fragrans | Kafr | Ericaceae | BERR | 2021 | BERR | 2003 |
| Leiophyllum buxifolium or Kalmia buxifolia | Lebu | Ericaceae | NCBG | 2021 | NCBG | 1993 |
| Liatris novae-angliae | Lino | Asteraceae |  |  | NPT | 1991 |
| Lilium parryi | Lipa | Liliaceae | CalBG | 2021 | CalBG | 1990 |
| Linum carteri var. carteri | Lica | Linaceae | FTBG | 2021 | FTBG | 2003 |
| Lomatium bradshawii | Lobr | Apiaceae | IAE | 2021 | BERR | 1990 |
| Lupinus westianus var. aridorum | Luwe | Fabaceae | BOK | 2021 | BOK | 2010 |
| Lycium sandwicense | Lysa | Solanaceae | NTBG | 2021 | NTBG | 2006 |
| Metrosideros polymorpha var. polymorpha | Mepo | Myrtaceae | HLA | 2022 | HLA | 1999 |
| Muhlenbergia microsperma | Mumi | Poaceae | SDZWA | 2024 | SDZWA | 2010 |
| Nolina brittoniana | Nobr | Agavaceae | BOK | 2021 | BOK | 1986 |
| Ornithostaphylos oppositifolia | Orop | Ericaceae | SDZWA | 2022 | CalBG | 1991 |
| Osteomeles anthyllidifolia | Osan | Rosaceae | HLA | 2021 | HLA | 2000 |
| Oxypolis canbyi | Oxca | Apiaceae | NCBG | 2022 | NCBG | 1988 |
| Packera franciscana or Senecio franciscanus | Pafr | Asteraceae | FLAG | 2022 | FLAG | 1991 |
| Penstemon clutei | Pecl | Scrophulariaceae | FLAG | 2021 | FLAG | 1991 |
| Penstemon peckii | Pepe | Scrophulariaceae | BERR | 2022 | BERR | 1992 |
| Penstemon shastensis | Pesh | Scrophulariaceae | CNPS | 2023 | CalBG | 1993 |
| Phacelia formosula | Phfo | Boraginaceae | DBG | 2021 | DBG | 1987 |
| Physaria globosa | Phgl | Brassicaceae | MBG | 2021 | MBG | 1995 |
| Physaria obcordata | Phob | Brassicaceae | DBG | 2022 | DBG | 1987 |
| Pinus radiata | Pira | Pinaceae |  |  | NLGRP | 2005 |
| Pityopsis ruthii | Piru | Asteraceae | NCBG | 2022 | NCBG | 1994 |
| Plagiobothrys hirtus | Plhi | Boraginaceae | IAE | 2021 | BERR | 1987 |
| Polemonium eddyense or Polemonium chartaceum | Poed | Polemoniaceae | CNPS | 2019 | NLGRP | 1991 |
| Polemonium occidentale ssp. lacustre | Pooc | Polemoniaceae | UMLA | 2021 | CBG | 1998 |
| Polyscias racemosa or Polyscias lallanii | Pora | Araliaceae | NTBG | 2021 | NTBG | 1999 |
| Ptilimnium nodosum | Ptno | Apiaceae | NCBG | 2022 | NCBG | 1987 |
| Purshia subintegra | Pusu | Rosaceae | FLAG | 2021 | FLAG | 1998 |
| Remirea maritima | Rema | Cyperaceae | FTBG | 2021 | FTBG | 2003 |
| Remya kauaiensis | Reka | Asteraceae | NTBG | 2022 | NTBG | 1990 |
| Rhus kearneyi ssp. kearneyi | Rhke | Anacardiaceae | DES | 2022 | DES | 1986 |
| Sarracenia oreophila | Saor | Sarraceniaceae | ABG | 2021 | NCBG | 1987 |
| Schoenoplectus tabernaemontani | Scta | Cyperaceae | NTBG | 2021 | NTBG | 2005 |
| Senecio ertterae | Seer | Asteraceae | IAE | 2021 | BERR | 1994 |
| Sesbania tomentosa | Seto | Fabaceae | NTBG | 2021 | NTBG | 1996 |
| Sidalcea nelsoniana | Sine | Malvaceae | IAE | 2021 | BERR | 1985 |
| Sisyrinchium sarmentosum | Sisa | Iridaceae | BERR | 2023 | BERR | 1996 |
| Solidago plumosa | Sopl | Asteraceae | NCBG | 2021 | NCBG | 2003 |
| Tephrosia angustissima var. corallicola | Tean | Fabaceae | FTBG | 2022 | FTBG | 2000 |
| Vaccinium boreale | Vabo | Ericaceae | NPT | 2023 | NPT | 1997 |
| Vaccinium crassifolium ssp. sempervirens | Vacr | Ericaceae | NCBG | 2021 | NCBG | 1988 |
| Warea amplexifolia | Waam | Brassicaceae | BOK | 2021 | BOK | 1988 |
| Ziziphus celata | Zice | Rhamnaceae | BOK | 2021 | BOK | 2008 |
